# Dual readout RT-LAMP for rapid on-site dengue virus detection and serotyping

**DOI:** 10.1128/spectrum.00040-26

**Published:** 2026-09-02

**Authors:** Rebecca Brambilla, Neesha Rockwood, Janine Michel, Kalpa Kariyawasam, Hansi Ganhewa, Johannes Scholz, Inoka C. Perera, Andreas Nitsche, Andreas Puyskens

**Affiliations:** 1 Robert Koch Institute, Centre for Biological Threats and Special Pathogens, ZBS 1: Highly Pathogenic Viruses, WHO Reference Laboratory for SARS-CoV-2 and WHO Collaborating Centre for Emerging Infections and Biological Threats 9222, Berlin, Germany; 2 Department of Medical Microbiology and Immunology, Faculty of Medicine, University of Colombo 58818, Colombo, Sri Lanka; 3 Department of Infectious Diseases, Imperial College London 4615, London, United Kingdom; 4 Department of Zoology and Environment Sciences, University of Colombo 58818, Colombo, Sri Lanka; National Chung Hsing University, Taichung, Taiwan

**Keywords:** molecular diagnostics, isothermal amplification, loop-mediated isothermal amplification, dengue serotyping, visual detection, point-of-care diagnostics

## Abstract

Dengue is the fastest-growing mosquito-borne disease, with over 14 million reported cases globally in 2024, rendering dengue a major public health issue of global concern. Fast and reliable serotype-specific diagnosis of dengue is critical for patient risk management and epidemiological surveillance. While real-time PCR currently remains the gold standard for dengue virus detection, it requires thermal cyclers and trained personnel in an established laboratory infrastructure, limiting its use in low-resource settings. Loop-mediated isothermal amplification (LAMP) offers a promising alternative, yet currently available reverse transcription assays for dengue either rely on limited sequences used for primer design, thus failing to adequately capture global genetic diversity, or use multiple primer sets, increasing complexity and costs. We developed four serotype-specific dengue virus (DENV) reverse transcription (RT)-LAMP assays in which primer design was informed by 4,277 whole-genome sequences from different geographical regions. Degenerate base positions were incorporated into primers to achieve broad detection capability while minimizing the number of distinct oligonucleotides needed. The assays achieved analytical sensitivities of 96–734 genome equivalents per reaction after 60 min. Clinical evaluation using 95 PCR-confirmed positive samples from Sri Lanka, including serotypes 1–3, demonstrated detection rates of 91.2% for DENV1, 78.6% for DENV2, and 100% for DENV3, with no cross-reactivity observed between serotypes. Evaluation using DENV4-spiked serum demonstrated reliable detection at clinically relevant viral loads, with complete detection at quantification cycle (Cq) values of ~30. A dual readout design enables both real-time fluorescence detection and equipment-free visual colorimetric detection, rendering the assays suitable for point-of-care applications in dengue-endemic regions worldwide.

**IMPORTANCE:**

Dengue virus infections pose a growing threat to global health, with cases doubling annually since 2021, yet reliable diagnostic tools remain inaccessible in many affected regions. Current gold-standard molecular tests require expensive equipment and specialized laboratories, creating a critical gap in dengue-endemic areas where rapid, on-site diagnosis could save lives through early patient management and outbreak control. This study addresses this gap by developing dengue virus serotype-specific detection assays that work at a constant temperature without the need for sophisticated and expensive equipment. By analyzing thousands of dengue virus genome sequences and strategically incorporating degenerate bases, we designed primers that can detect diverse viral strains while maintaining simplicity and low cost. The assays provide results in 40–60 min, depending on the workflow, and can be read visually without instruments, making them practical for use in clinics, field settings, and resource-limited laboratories where dengue burden is highest.

## INTRODUCTION

Dengue is the fastest-growing mosquito-borne disease with over 14 million reported cases worldwide in 2024 (1). The number of dengue cases has approximately doubled each year since 2021, with surges across all six World Health Organization regions (2). Dengue virus (DENV), the causative pathogen of dengue, is currently endemic in over 100 countries, with major endemic regions encompassing South America, South Asia, and Africa (3–5). DENV is a single-stranded ribonucleic acid (RNA) virus that belongs to the genus *Orthoflavivirus* (formerly *Flavivirus*) and is a member of the family *Flaviviridae*. To date, there are four known antigenically distinct DENV serotypes reported: DENV1, DENV2, DENV3, and DENV4. DENV is transmitted through bites of mosquitoes of the *Aedes* genus, with *Aedes aegypti* and *Aedes albopictus* as the major species causing DENV transmission in humans (6, 7). DENV infection leads mostly to mild, flu-like illness with nonspecific symptoms. However, in some cases, DENV infection can progress to severe disease, such as dengue hemorrhagic fever or dengue shock syndrome (6). Previous studies indicate that patients with secondary dengue infections caused by heterologous serotypes show an increased risk of developing severe dengue compared to primary infections, potentially through antibody-dependent enhancement (8–10). Importantly, all DENV serotypes can induce severe disease. This underlines the importance of DENV serotyping for understanding disease severity and epidemiological surveillance. Serotype distribution data are crucial for deciding where and how vaccines are rolled out and for evaluating vaccine effectiveness by serotype (11, 12).

Dengue is commonly diagnosed either by detection of the NS1 antigen or the detection of viral RNA by real-time reverse transcription polymerase chain reaction (rtRT-PCR). While antigen detection is widely used to diagnose acute DENV infection, currently it cannot be used for serotyping (13). In contrast, rtRT-PCR enables specific and sensitive detection of viral RNA even during early stages of infection and can be used to perform serotyping (14, 15). While rtRT-PCR is the current gold standard in the diagnosis of dengue, it relies on the use of real-time thermal cyclers and testing conducted by trained personnel, typically in a laboratory setting. These factors negatively impact the applicability of rtRT-PCR in remote and low-resource settings (16).

Loop-mediated isothermal amplification (LAMP) is an isothermal amplification technique that, like real-time PCR, offers high specificity and sensitivity, omitting the need for expensive real-time thermal cyclers while delivering simplified workflows with enhanced detection speeds. Hence, LAMP offers a promising alternative for the diagnosis of dengue in settings where rtRT-PCR is not available (16, 17).

Several reverse transcription (RT)-LAMP assays have been designed for the detection and serotyping of DENV (18). For the majority of already published RT-LAMP assays, the number of DENV sequences used for primer design is either not disclosed or only a very limited number of DENV sequences were used for primer design (16, 19–27), which does not adequately reflect present global DENV genetic diversity. Only Lopez-Jimena et al*.* present a comprehensive data set of DENV whole-genome sequences used for primer design (28). However, to address DENV serotype genetic diversity, the authors use 37 individual LAMP primer sets in one mix, which increases the probability of primer-primer interactions, preparation complexity, as well as cost.

In this study, we designed rapid and sensitive RT-LAMP assays for specific detection of DENV serotypes, informed by 4,277 DENV whole-genome sequences from across different geographical regions. Degenerate base positions were used to address the genetic diversity of different dengue virus strains, while reducing the number of distinct oligonucleotides that need to be added to the reaction mix. Analytical sensitivities (limit of detection with 95% probability) were determined as 96 DENV genome equivalents (ge)/reaction for DENV1, 734 ge/reaction for DENV2, 187 ge/reaction for DENV3, and 473 ge/reaction for DENV4, with assay run times under 60 min. Clinical evaluation in Sri Lanka using 95 PCR-confirmed DENV-positive samples, including DENV serotypes 1–3, demonstrated RT-LAMP detection rates of 91.2% for DENV1, 78.6% for DENV2, and 100% for DENV3, with no cross-reactivity observed between serotypes. DENV4 performance was confirmed using spiked serum samples, demonstrating reliable detection at clinically relevant concentrations. A dual readout design enables both real-time fluorescence detection and equipment-free visual colorimetric detection. The presented assays offer an affordable, rapid, and sensitive solution for on-site DENV detection and serotyping when rtRT-PCR is not available.

## MATERIALS AND METHODS

### DENV cell culture and clinical specimens

DENV serotypes 2, 3, and 4 were obtained from the American Type Culture Collection (ATCC): DENV2 (VR345), DENV3 (VR216), DENV4 (VR217). DENV1 was kindly provided by the Bernhard Nocht Institute for Tropical Medicine (BNITM), Hamburg, Germany. DENV serotypes were cultured on VeroE6 cells (#85020206, ECACC). Cell culture supernatants were chemically inactivated using AVL (QIAGEN) and ethanol. Sample extraction was performed using the QIAamp Viral RNA Mini Kit (QIAGEN) according to the manufacturer’s instructions. Sample extracts were eluted in 60 µL nuclease-free H_2_O. Samples were kept briefly at 4°C before testing and were subsequently stored at −80°C.

Between January 2022 and May 2023, participants were recruited as part of a larger study assessing epidemiology and diagnostics for acute undifferentiated febrile illness from three government-run district general hospitals in the Kalutara District, Western Province, and Nawalapitiya and Gampola, Kandy District, Central Province, Sri Lanka. These hospitals were chosen as admissions typically represent early community-onset febrile illness. Eligible participants were ≥14 years old, with fever ≥38°C for ≤7 days without a clear focus of infection and requiring admission. Exclusion criteria included >2 days of prior antibiotics, nosocomial or postoperative infections, trauma, chemotherapy, or immunosuppression. Blood was collected, and serum was separated from blood at the clinical site. Clinical samples were transported on ice to the Department of Microbiology, University of Colombo. For this study, a cohort of 95 DENV PCR-positive study participants was included in the analyses. Of the 95 patients, 64 (67%) were male, with a median age of 30 years (IQR 22.5–44.5). The median duration of fever at the time of sampling was 4 days (range 1–7 days). NS1 antigen positivity was observed in 88/95 (93%) and dengue IgM seropositivity in 44/95 (46%) of cases. Clinical diagnosis of dengue was only correctly made in 64/95 (67%) of cases. Additionally, 68 PCR-confirmed DENV-negative serum samples from chikungunya and 10 samples from leptospirosis patients from Sri Lanka were included to assess clinical specificity. For RT-LAMP and rtRT-PCR analysis, analogous to samples obtained from cell culture supernatants, clinical specimens were inactivated and extracted using the Viral RNA Mini Kit (QIAGEN) and eluted in 60 µL nuclease-free H_2_O. Samples were kept briefly at 4°C before testing and were subsequently stored at −80°C.

### Virus quantification by real-time PCR and digital PCR

In-house DENV generic (DENV all) and serotype-specific (DENV1–4) rtRT-PCR assays were used for quantification and serotyping of samples and clinical specimens (Table 1). The rtRT-PCR assays were performed using 3 µL template in a total reaction volume of 25 µL using the AgPath-ID One-step RT-PCR kit (Applied Biosystems, Thermo Fisher Scientific) on a CFX96 Touch Real-Time PCR Detection System (BioRad).

**TABLE 1:** Primer sequences of DENV real-time PCR assays^*a*^

Primer name	Primer sequence	Target region
DENV all R	CgYTCTgTgCCTggAWTgATg	3′UTR
DENV all F	AAACAgCATATTgACSCTggg	
DENV all TM	6-FAM-AgAC(+C)AgAgATCCTg(+C)TgTCT-BHQ-1	
DENV1-2F	CAANCCATggAAgCTgTACgC	3′UTR
DENV 3F	CAAACYgTggAAgCTgTACgC	
DENV 4F	gCCTCggAAgCTgTACgC	
DENV1-4R	CCCAgSgTCAATATgCTgTTT	
DENV1 TM	FAM-CACCAKggRAAgCTgTAYCYTggTggT-BHQ1	
DENV2 TM	YAK-CCCAAggYgAgATgAAgCTgTAgTCTCRCTg-BHQ1	
DENV3 TM	ROX-ACTgAggAAgCTgYACCTCCYTgCA-BHQ2	
DENV4 TM	CY5-CAggAggAAgCTgTACTYCTggTggAAg-BHQ3	

^*a*^ R, reverse primer; F, forward primer; TM, TaqMan probe; BHQ1/2/3, Black Hole Quencher 1/2/3; FAM, fluorescein amidites fluorophore; YAK, Yakima yellow; ROX, 6-carboxy-X-rhodamine; Cy5, Cyanine5; +C, locked nucleic acid (LNA)-modified cytosine; UTR, untranslated region. Primer binding region relative to NC_001477 (DENV1), NC_001474 (DENV2), NC_001475 (DENV3), NC_002640 (DENV4).

Digital (d)PCR was used for absolute quantification of viral RNA with the QIAcuity Digital PCR System (QIAGEN). DENV all primers and probes were used with the QIAcuity OneStep Advanced Probe Kit (QIAGEN) according to the manufacturer’s instructions. Specifically, 3 µL of template was used in a total reaction volume of 40 µL in a 26k 24-well nanoplate (QIAGEN). Viral concentrations are shown as genome equivalents (ge)/reaction.

### RT-LAMP assay design

RT-LAMP assays were designed for serotype-specific detection of DENV. For primer design, all available whole-genome sequences for DENV1 (1,790 sequences), DENV2 (1,290 sequences), DENV3 (907 sequences), and DENV4 (290 sequences) were obtained from NCBI Virus Variation DENV Database (29) (accessed 4 July 2023) and aligned using MAFFT (30). Alignments were screened for regions of highest genetic conservation within serotypes, and candidate regions were selected for primer design in PrimerExplorer v5 (31) using default LAMP parameters. To maximize genetic coverage across circulating DENV strains, degenerate bases were incorporated at positions with ≤95% sequence homology. Candidate primer sets were first filtered *in silico* based on Tm compatibility, absence of cross-reactivity between primers, and BLASTn (32, 33) specificity against human sequences, other orthoflaviviruses, and common differential diagnosis pathogens. Subsequently, remaining candidates were evaluated *in vitro* for assay sensitivity, speed, and specificity.

### Dual readout RT-LAMP

RT-LAMP assays were optimized for dual readout, enabling real-time detection using the NEB LAMP Fluorescent Dye (NEB; B1700S) and colorimetric endpoint detection using the WarmStart Colorimetric LAMP 2× Master Mix (NEB; M1800). A single 25 μL RT-LAMP reaction contained 3 μL template, 12.5 μL 2× Master Mix, 0.5 μL 50× Fluorescent Dye, 2.5 μL 10× primer mix (final concentrations: F3/B3 0.2 μM each; FIP/BIP 1.6 μM each; LF/LB 0.4 μM each), and nuclease-free H_2_O. For RT-LAMP assay optimization, specificity and sensitivity evaluations, testing was performed in 96-well PCR plates on a CFX96 Touch Real-Time PCR Detection System (BioRad) at a constant temperature of 65°C for 60 min. Time-to-result in minutes represents the time for the fluorescence signal to reach the detection threshold. Fluorescence was detected in the SYBR GREEN/FAM channel (excitation: 463–503 nm, emission: 518–548 nm), and colorimetric readout was performed visually. Samples were noted positive when the fluorescence signal reached the threshold and the colorimetric reaction resulted in a color shift of the reaction mix from pink to yellow when compared to the positive and negative controls. To minimize the risk of amplicon carryover contamination, pre- and post-amplification work was performed in physically separated areas using dedicated pipettes and consumables. No template controls (NTCs) were included in every experimental run to monitor for contamination. Importantly, both readout modalities employ dyes that are incorporated into the reaction mix prior to amplification, enabling signal detection through sealed tubes without post-amplification tube opening. This closed-tube format eliminates the need for post-amplification addition of indicator dyes and thereby minimizes the risk of amplicon carryover contamination.

### Optimization of RT-LAMP reaction for on-site testing with the Axxin T8-ISO

RT-LAMP protocols were optimized for use in the T8-ISO isothermal amplification instrument (Axxin). Each RT-LAMP reaction was performed on a MicroAmp 8-Tube Strip (Applied Biosystems). A single 25 µL RT-LAMP reaction contained 3 µL template, 12.5 µL 2× Master Mix, 1.25 µL of 5× Fluorescent Dye, 2.5 µL 10× primer mix (final concentrations: F3/B3 0.2 µM each; FIP/BIP 1.6 µM each; LF/LB 0.4 µM each), and nuclease-free H_2_O. Reaction time was adjusted to 40 min at a constant temperature of 65°C.

### Specificity testing

DENV1–4 RT-LAMP assays were tested *in vitro* for cross-reactivity with viral pathogens relevant for differential diagnosis, including chikungunya virus (CHIKV), influenza A-3361 (IFV A-3361) and influenza A-3340 (IFV A-3340), measles virus (MV), West Nile virus (WNV), yellow fever virus (YFV), Zaire ebolavirus (EBOV Zaire), and Zika virus (ZIKV). Cross-reactivity with CHIKV was assessed using rtRT-PCR-confirmed CHIKV-positive, DENV-negative clinical serum extracts from Sri Lanka (*n* = 68); all other pathogens were tested using extracts from cell culture-derived virus. Sequencing of LAMP amplicons for DENV1–4 by MinION (Oxford Nanopore Technologies) was performed to confirm RT-LAMP target specificity. Sequencing library preparation was performed using the Rapid Barcoding Kit 24 V14 (Oxford Nanopore Technologies) according to the manufacturer’s instructions.

### Determination of analytical sensitivity

To determine analytical sensitivities of DENV RT-LAMP assays, probit analyses were performed using 1:4 serial dilutions of DENV RNA quantified by dPCR. In total, twelve replicates per dilution were tested for each of the DENV RT-LAMP assays. The limit of detection (LOD_95%_) represents the concentration of viral RNA that can still be detected in the LAMP assay with 95% probability. LOD_95%_ was calculated using nonlinear regression in GraphPad Prism (version 9.1.0).

### Molecular testing of clinical specimens in Sri Lanka

Patient sera were initially tested for DENV using the serotype-specific DENV rtRT-PCRs (Table 1). In total, extracts from 95 rtRT-PCR-confirmed DENV-positive sera and 78 DENV-negative sera from leptospirosis (*n* = 10) and chikungunya (*n* = 68) patients were used to assess the clinical performance of the DENV RT-LAMP assays. Extracts of chikungunya patients were diluted 1:4 in nuclease-free H_2_O prior to testing due to limited availability in sample volume. Each sample was tested in all four DENV RT-LAMP assays. Time-to-result based on fluorescence was determined, and colorimetric results were noted. Testing was performed on a CFX96 Touch Real-Time PCR Detection System (Bio-Rad).

Due to the absence of DENV4 among clinical specimens from Sri Lanka, DENV4 RT-LAMP performance was evaluated using spiked serum samples. Pooled DENV-negative serum from healthy controls was spiked with cell culture-derived DENV4 at defined concentrations. Three independent extractions were performed per concentration using the QIAamp Viral RNA Mini Kit (140 µL serum input, 60 µL elution volume), analogous to the extraction procedure used for clinical specimens. Eluates were tested in parallel by rtRT-PCR and DENV4 RT-LAMP. Additionally, a subset of DENV4-spiked serum extracts (*n* = 3) was tested in all four DENV RT-LAMP assays to assess inter-assay cross-reactivity.

## RESULTS

### RT-LAMP assay design

**Fig 1:**
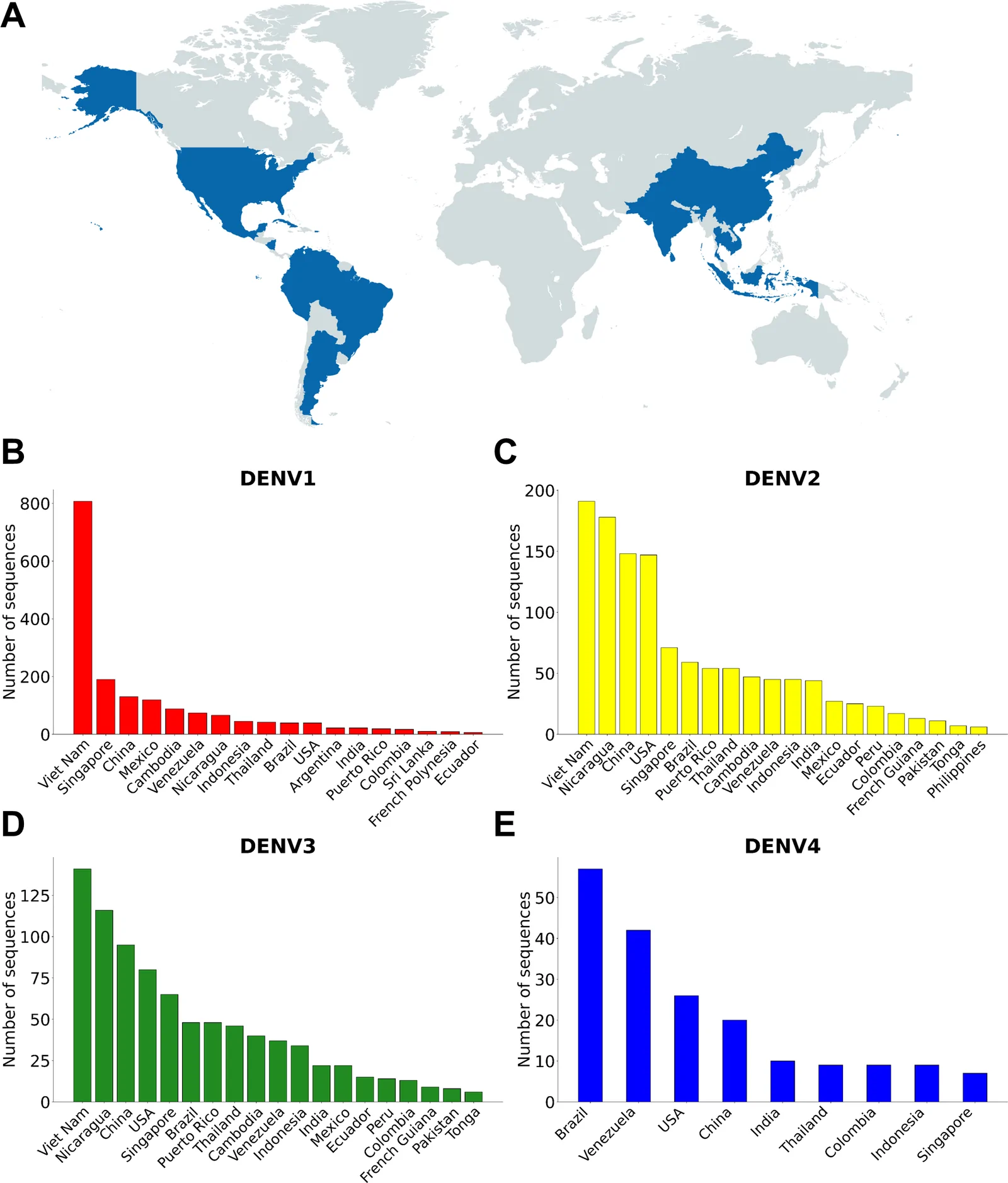
Graphical overview of origin diversity in sequences used in primer design process. (**A**) Map showing the geographical origin of DENV whole-genome sequences used for primer design (created with mapchart.net). (**B–E**) Overview of the number of sequences from respective countries used in serotype-specific sequence alignments.

In total, 4,277 publicly available DENV whole-genome sequences from different geographical regions were included in primer design (Fig. 1). To address the large genetic variability among DENV serotypes, while at the same time keeping the number of distinct oligonucleotides required for successful LAMP reaction at a minimum, degenerate base positions were incorporated into the primer design. After initial functionality testing and *in silico* specificity evaluation, the best performing primer sets for each serotype were selected for further performance evaluation. Best performing primer sets and their target regions are listed in Table 2.

**TABLE 2:** Serotype-specific DENV RT-LAMP assays^*a*^

LAMP primer	Primer sequence	Target region (binding region)
F3-DENV1	TggACYCTCTATgCAgTgg	Nonstructural protein NS4B (nt 6,941–7,138)
B3-DENV1	RTTCACCTgSgAATAgCACC	
FIP-DENV1	ggTTTgCAATRgCTgTCAgggAACTCCCATgATgAggCACA	
BIP-DENV1	TgggACTTgACAAAggATggCCYAAggCgAgAAgTggAACYC	
LF-DENV1	TgTTTgCCgTTgTRTTTTCRAT	
F3-DENV2	CgAgTRgARATgggTgARg	Nonstructural protein NS3 (nt 5,428–5,609)
B3-DENV2	ggAACRAACCAHACAgTCTT	
FIP-DENV2	TgCTYTgRggAAATggRTCTCAgCYgggATTTTYATgACA	
BIP-DENV2	AYgCACCAATCATRgATgARgAACCCTTYAAARTCCgTRACC	
LB-DENV2	CCTgARCgTTCRTggAATTCWg	
LF-DENV2	TRCTTCCMggRggAgTggC	
F3-DENV3	TgCTgAAACgCgTgAgAA	Capsid protein C(nt 138–350)
B3-DENV3	TCTTgAAgCCTYTYAggACC	
FIP-DENV3	CYTggCCRTTCAgCAATCCTTTCCgTgTgTCAACTggATCAC	
BIP-DENV3	RgCCATTCCACCRACAgCAggTRATRgCCCCYgACTTCT	
LB-DENV3	TTggCYAgATggggAACCTT	
F3-DENV4	gATgTCATCggAAgRggC	Membrane glycoprotein M – envelope protein E(nt 770–989)
B3-DENV4	ACCYgAgACTCCTTCYACAA	
FIP-DENV4	TgCCARgAgYgCRAATCCTggTggAARCAYgCYCAgAgRgT	
BIP-DENV4	TYgggCAAACAggRATCCAgCgACgCATCgCATTCCgTAR	
LB-DENV4	AATgATgCTRgTCgCYCC	

^*a*^ A, adenine; C, cytosine; g, guanine; T, thymine; R, A or G; Y, C or T; S, G or C; W, A or T; M, A or C; H, A or C or T. Nucleotide (nt); LAMP primer sets binding region relative to NC_001477 (DENV1), NC_001474 (DENV2), NC_001475 (DENV3), and NC_002640 (DENV4).

### Specificity of DENV RT-LAMP assays

**Fig 2:**
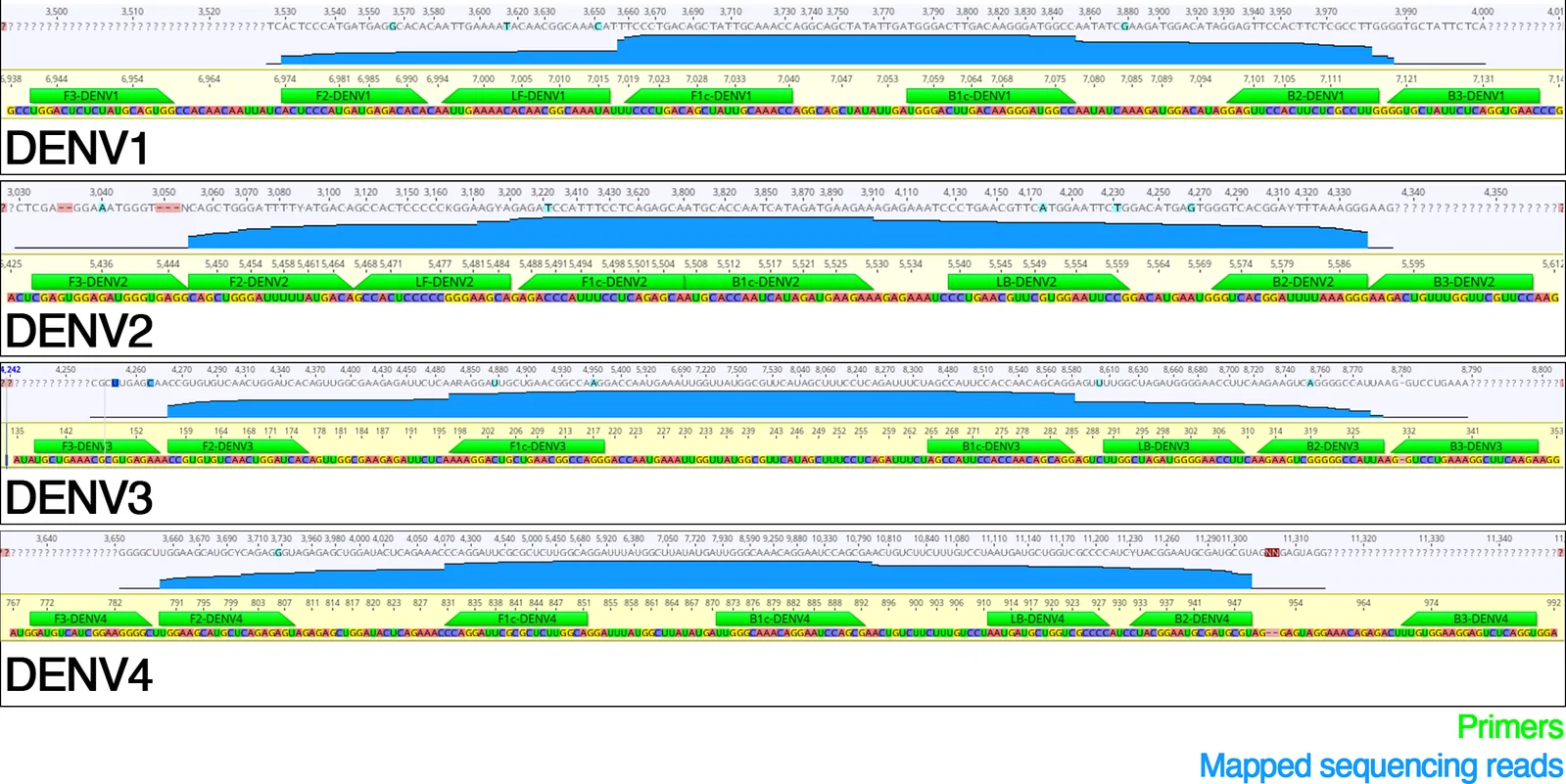
Binding of DENV1–4 RT-LAMP primer sets and mapping of amplicon sequencing reads to the primer target region. Mapped reads: DENV1 = 2,578; DENV2 = 6,965; DENV3 = 42,216; DENV4 = 40,807.

**Fig 3:**
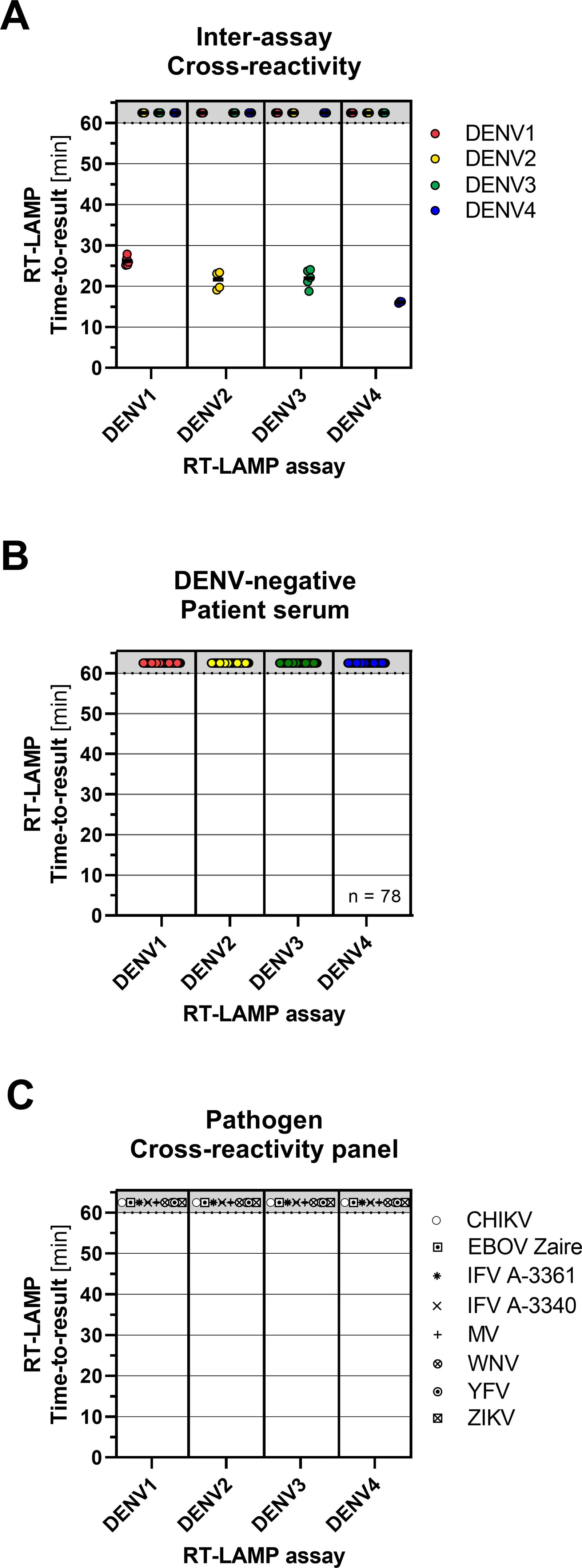
Specificity evaluation of DENV1–4 RT-LAMP assays. Gray shaded areas indicate negative LAMP results (no amplification within 60 min). Dotted line represents assay cut-off time at 60 min. (**A**) Inter-assay cross-reactivity: a subset of DENV1 (*n* = 5), DENV2 (*n* = 5), and DENV3 (*n* = 5) clinical specimens with Cq values ranging between 22 and 30, and DENV4-spiked serum samples (*n* = 3, Cq 20–21) were tested in all four DENV RT-LAMP assays. (**B**) Extracts from PCR-confirmed DENV-negative sera (*n* = 78) from Sri Lanka were tested in all four DENV RT-LAMP assays. No false-positive results were observed. (**C**) RT-LAMP assays tested against CHIKV, EBOV Zaire, IFV A-3361, IFV A-3340, MV, WNV, YFV, and ZIKV. No cross-reactivity was observed.

Sequencing of LAMP amplicons confirmed that amplification products are specific to the respective genomic regions targeted by the LAMP primers (Fig. 2). Furthermore, cross-reactivity testing using DENV1–3 clinical specimens (*n* = 5 per serotype, Cq 22–30) and DENV4-spiked serum samples (*n* = 3, Cq 20–21) tested in all four DENV RT-LAMP assays showed no cross-reactivity between serotypes, with each sample detected exclusively by the respective serotype-specific assay among the specimens tested (Fig. 3A). Additionally, 78 PCR-confirmed DENV-negative sera from chikungunya (*n* = 68) and leptospirosis (*n* = 10) patients tested negative in all four RT-LAMP assays, with no false-positive results observed (Fig. 3B). Lastly, none of the RT-LAMP assays displayed cross-reactivity with any of several viral pathogens relevant for differential diagnosis (Fig. 3C).

### Analytical sensitivity of DENV RT-LAMP assays

**Fig 4:**
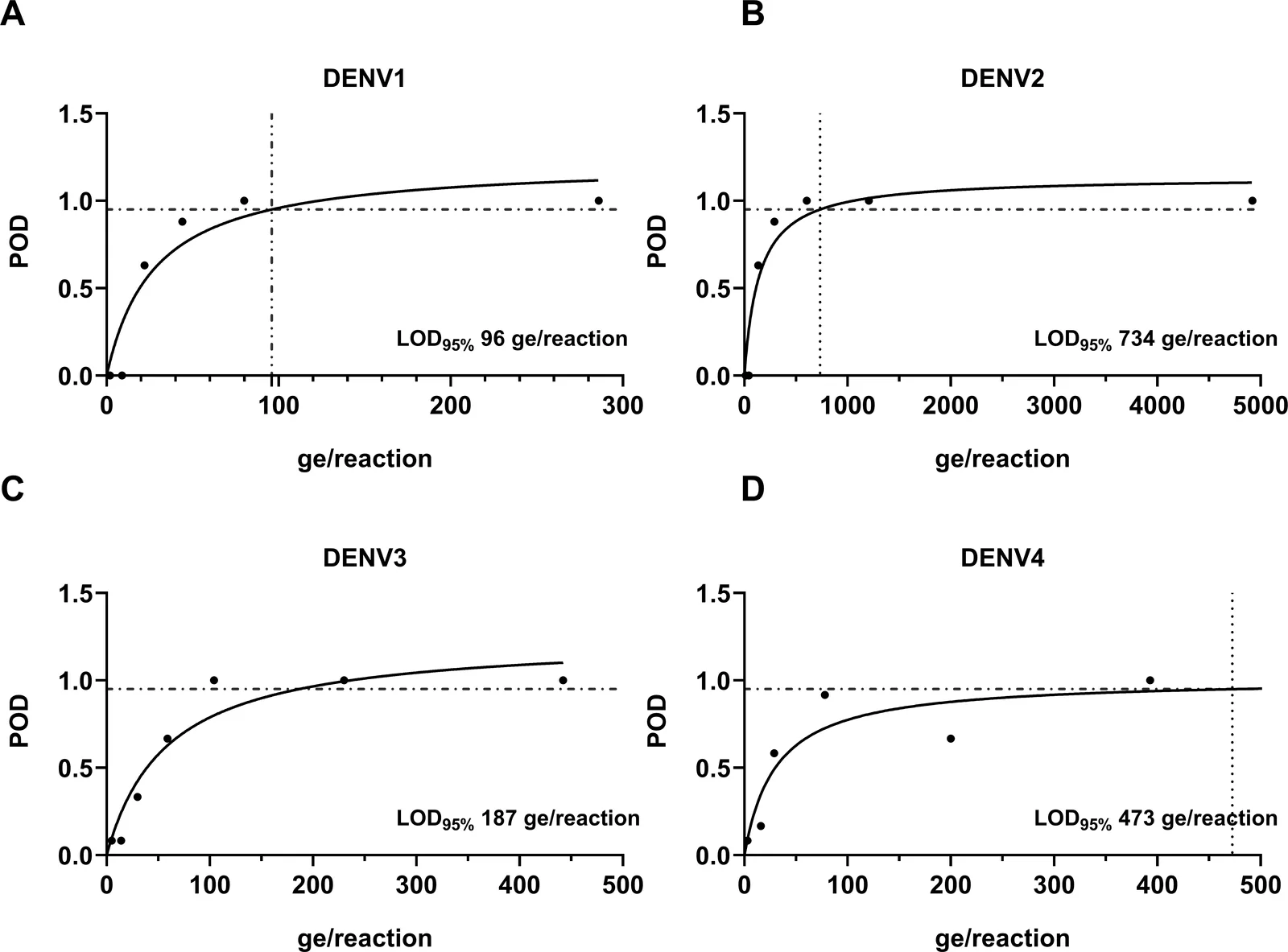
Analytical sensitivity of DENV1–4 RT-LAMP assays as determined by probit analyses. Correlation between probability of detection (POD) and DENV genome equivalents (ge)/reaction. Twelve replicates per dilution were tested. (**A**) DENV1 LOD_95%_ = 96 ge/reaction, (**B**) DENV2 LOD_95%_ = 734 ge/reaction, (**C**) DENV3 LOD_95%_ = 187 ge/reaction, and (**D**) DENV4 LOD_95%_ = 473 ge/reaction.

Probit analyses were used to assess the analytical sensitivity of the DENV RT-LAMP assays. LOD_95%_ varied between serotypes, with 96 ge/reaction for DENV1, 734 ge/reaction for DENV2, 187 ge/reaction for DENV3, and 473 ge/reaction for DENV4 (Fig. 4).

### Performance of DENV RT-LAMP assays with clinical specimens

**Fig 5:**
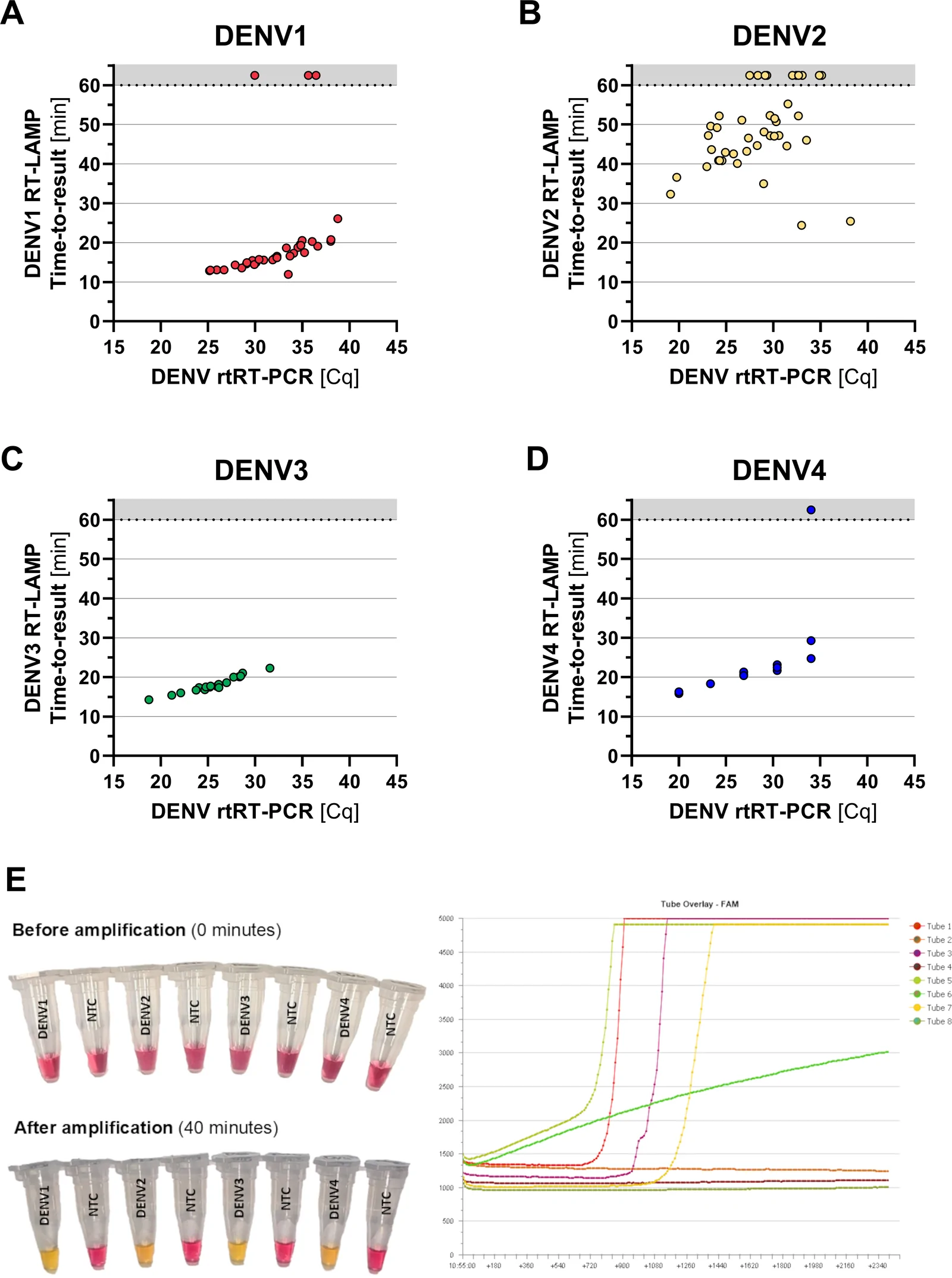
Clinical performance of serotype-specific DENV RT-LAMP assays and point-of-care feasibility. Gray shaded areas indicate negative LAMP results (no amplification within 60 min). Dotted line represents assay cut-off time at 60 min. Each data point represents an individual sample. Correlation between reference rtRT-PCR Cq values and RT-LAMP time-to-result (min) for (**A–C**) clinical serum specimens from Sri Lanka and (**D**) DENV4 RT-LAMP: spiked serum samples at defined concentrations, with three independent extractions per concentration. (**A**) DENV1 RT-LAMP detection of 31/34 (91.2%) PCR-positive DENV1 specimens. (**B**) DENV2 RT-LAMP detection of 33/42 (78.6%) PCR-positive DENV2 specimens. (**C**) DENV3 RT-LAMP detection of 19/19 (100%) PCR-positive DENV3 specimens. (**D**) DENV4 RT-LAMP detected all samples with Cq values ≤30.4, achieving 100% detection (3/3) down to an average serum concentration of 2.68 × 10³ ge/mL. At the lowest concentration tested (average 325 ge/mL, Cq ~34.0), the assay detected 2/3 samples. (**E**) Detection with the battery-operated Axxin T8-ISO portable isothermal amplification instrument using dual readout with optimized protocol (0.25× fluorescent dye and reduced gain in the FAM test channel to 7%). Colorimetric change before and after amplification reaction: positive results show a shift to yellow, negative results remain pink. DENV-positive samples show an increase in fluorescence, which is not observed in the NTC. DENV1 (Tube 1), DENV2 (Tube 3), DENV3 (Tube 5), DENV4 (Tube 7), NTC (Tubes 2, 4, 6, and 8).

A total of 95 DENV PCR-positive patient sera were tested using the developed RT-LAMP assays at the Department of Medical Microbiology and Immunology at the Faculty of Medicine, University of Colombo, Colombo, Sri Lanka. Serotype-specific rtRT-PCR determined the distribution of DENV-positive sera as DENV1 (*n* = 34), DENV2 (*n* = 42), and DENV3 (*n* = 19), with no DENV4 detected among the tested clinical specimens. RT-LAMP assays for the detection of DENV1 and DENV3 demonstrated a pronounced correlation between rtRT-PCR Cq values and RT-LAMP time-to-result, with faster amplification times generally observed for specimens with lower Cq values (higher viral loads) (Fig. 5A and C). For the DENV2 RT-LAMP assay, a moderate correlation was observed, with greater variation in detection times even among specimens with similar Cq values (Fig. 5B). The DENV1 RT-LAMP assay detected 31/34 (91.2%) of the PCR-positive DENV1 specimens tested, with detection times ranging from approximately 12–26 min (Fig. 5A). The DENV2 RT-LAMP assay detected 33/42 (78.6%) of DENV2-positive specimens, with detection times predominantly between 30 and 55 min and overall higher variability between specimens than the other serotypes (Fig. 5B). The DENV3 RT-LAMP assay detected all 19/19 (100%) DENV3-positive specimens, with detection times ranging from 15 to 22 min (Fig. 5C). Overall, 83/95 (87.4%) of DENV PCR-positive specimens were correctly detected by the RT-LAMP assays. Among the twelve clinical specimens not detected by RT-LAMP, nine were DENV2-positive. Of these, six samples had Cq values >30 and the remaining three samples had Cq values >28. Likewise, the three specimens missed by DENV1 RT-LAMP had Cq values of ≥30. Due to the absence of DENV4 among clinical specimens, assay performance was evaluated using a serial dilution of serum spiked with cell culture-derived DENV4. The DENV4 RT-LAMP assay detected all samples with Cq values ≤30.4, achieving 100% detection (3/3) down to an average serum concentration of 2.68 × 10³ ge/mL. At the lowest concentration tested (average 325 ge/mL, Cq ~34.0), the assay detected 2/3 samples (Fig. 5D).

### Optimization of DENV RT-LAMP assays for point-of-care testing

To demonstrate the suitability of the developed RT-LAMP assays for point-of-care testing, DENV RT-LAMP assays with dual real-time fluorescence and colorimetric endpoint readout were optimized for use in the Axxin T8-ISO, a portable isothermal amplification instrument that can be battery-operated. As fluorescent dye concentrations used in the standard LAMP assays on real-time PCR fluorimeters would saturate the T8-ISO detector, we reduced fluorescent dye concentrations from 1× to 0.25× final concentration and adjusted the gain level of the FAM channel to 7%. Testing with DENV RT-LAMP assays on the Axxin T8-ISO showed clear distinction between positive and negative samples in both fluorescent real-time detection and colorimetric endpoint detection (indicated by a color shift of the reaction mix from pink to yellow for positive samples) (Fig. 5E).

## DISCUSSION

In this study, we present serotype-specific RT-LAMP assays for rapid DENV detection and serotype identification that demonstrate robust analytical and clinical performance suitable for point-of-care testing. The DENV RT-LAMP assays achieved analytical sensitivities of 96–734 ge/reaction. Performance with clinical specimens demonstrated detection rates of 78.6%–100% using PCR-confirmed DENV-positive patient sera in Sri Lanka.

The primer design strategy employed here differs from existing RT-LAMP assays for DENV detection in two key aspects. First, 4,277 publicly available DENV whole-genome sequences from different geographical regions were used for primer design, addressing a limitation of previous studies that relied on smaller or undisclosed data sets (16, 19–27). Second, strategic incorporation of degenerate bases enables broad-spectrum detection while minimizing the number of distinct oligonucleotides required for amplification. This contrasts with previous sequence-informed approaches that require a multitude of distinct oligonucleotides to achieve comparable genetic coverage (28), thereby increasing assay complexity and costs.

The analytical sensitivities achieved by the presented assays are either slightly lower or comparable with previously reported molecular point-of-care methods for DENV detection (16, 22, 26–28). Though, it is important to note that direct comparison of reported LOD values with previous publications is difficult, due to significant methodological heterogeneity across published DENV RT-LAMP assays. Several previous studies reporting lower detection limits employed plasmid DNA standards rather than viral RNA (22), quantified targets in plaque-forming units rather than genome copies (19), or determined LODs by endpoint dilution without probit analysis (21, 24), all of which can lead to apparent sensitivities that are substantially lower than probit-derived LOD_95%_ values determined with dPCR-quantified genomic RNA as employed here. Even among studies using replicate testing, reported values can be misleading: for example, Teoh et al. report detection of “up to 10 copies” but achieved only 25% positivity (3/12 replicates) at this concentration and 75% at 60 copies (26), data that would yield a probit-derived LOD_95%_ comparable to the values presented here. When compared to studies using methodology similar to ours, the achieved sensitivities are comparable to previously reported values (28). Expressed as serum concentrations, the here achieved LOD_95%_ correspond to approximately 1.4 × 10⁴ to 1.0 × 10⁵ ge/mL, assuming a complete extraction efficiency. These detection thresholds are well below typical viremia levels during the acute febrile phase, which multiple studies have consistently reported in the range of 10⁶ to 10⁹ copies/mL, with peak levels observed during the first 3 days after symptom onset (34–36). A pooled analysis of 5,642 dengue cases demonstrated that higher febrile-phase viremia is associated with adverse clinical outcomes regardless of serotype or immune status (37), indicating that these higher-risk patients carry viral loads that exceed the here presented assay detection limits by several orders of magnitude. While further improvements in analytical sensitivity are useful and could extend the detection window into later stages of infection when viremia declines, the achieved sensitivities are well-suited for detecting acute dengue infections during the febrile phase when molecular diagnosis is most clinically actionable.

Evaluation with clinical samples likewise showed robust overall performance, with notable differences in detection performance between serotypes: DENV1 (91.2%), DENV2 (78.6%), and DENV3 (100%). The reduced performance for DENV2, coupled with greater variability in detection times, is likely reflected by the highest level of primer degeneracy in the DENV2 RT-LAMP assay (13.6% degenerate base positions vs 5.6%–10.9% for other serotypes). In fact, analytical sensitivity ranked in the same order as primer degeneracy across all four serotypes. This is consistent with previous reports showing that increased primer degeneracy can reduce sensitivity due to decreased primer-template matching efficiency (38). The strong correlation observed between Cq values of the rtRT-PCR and RT-LAMP detection times for DENV1 and DENV3 in clinical samples, versus the more variable relationship observed for DENV2, seems to support this hypothesis. Increasing primer concentrations of the DENV2 assay could potentially improve assay performance. Furthermore, increasing template input volume may improve detection of low-titer specimens, particularly in settings where DENV2 predominates. Alternatively, sensitivity of the DENV2 RT-LAMP assay could likely be improved through a reduction in primer degeneracy; however, this would result in a reduction in viral genetic diversity that can be covered by the assay.

Due to the absence of DENV4 from our set of clinical specimens, we were unable to evaluate performance for DENV4 RT-LAMP assay with actual clinical specimens. The absence of DENV4 is in line with the epidemiological situation in Sri Lanka, where DENV4 circulation has been minimal in recent years (39, 40). To address this limitation, we evaluated DENV4 RT-LAMP performance using serum spiked with cell culture-derived DENV4, demonstrating reliable detection at Cq values below ~30, consistent with the detection performance observed for DENV1–3 clinical specimens. With respect to specificity, inter-serotype specificity was established on a defined panel of samples, in which each sample was amplified exclusively by its cognate serotype-specific assay, and supported by *in silico* screening against 4,277 DENV genomes, with amplicon sequencing confirming target-specific product identity. While no inter-serotype cross-reactivity was observed, rare cross-reactivity with strains not represented in this panel cannot be formally excluded; however, because each primer set targets a serotype-discriminating region that is conserved within its cognate serotype but divergent between serotypes, and because LAMP requires the coordinated binding of multiple primers in defined orientation and spacing, the compounding of mismatches across these sites renders cross-serotype amplification mechanistically unlikely. Likewise, no cross-reactivity was observed with the differential-diagnosis pathogens tested.

Compared to rtRT-PCR and NS1 rapid tests, the presented RT-LAMP assays offer several advantages for point-of-care applications (Table 3): (i) isothermal amplification eliminates the need for thermal cycling equipment, (ii) reduced assay run times, and (iii) visual readout capability. Alternatively, antigen testing, such as the NS1 ELISA, also has been reported to deliver sensitive and rapid results; however, it currently cannot provide serotype information (13, 41). While RT-LAMP assay run times of under 60 min are shorter compared to conventional rtRT-PCR, a major time-reducing benefit is the ability for point-of-care testing in LAMP. In lower-resource settings, such as in Sri Lanka, total turnaround time for rtRT-PCR results is typically extended by hours to days due to sample transport and batch processing in a centralized laboratory setting. RT-LAMP deployed at the point of care could eliminate these delays, providing actionable serotype-specific results within a clinically relevant timeframe.

**TABLE 3:** Comparison of dengue diagnostic approaches relevant to point-of-care use^*a*^

Method	Serotype information	Nucleic acid extraction	Equipment/infrastructure	Time from sample to result	Relative cost per test	Sensitivity in secondary infection
NS1 rapid test	No	Not required	Minimal (lateral flow)	~15–20 min	$	Reduced
Pan-DENV RT-LAMP (16)	No	Required	Low (heat-source/portable isothermal device)	Extraction + ~30–60 min	$$	High
Serotype-specific RT-LAMP (this study)	Yes (DENV1–4)	Required	Low (heat-source/portable isothermal device)	Extraction + 40–60 min	$$	High
rtRT-PCR	Yes (DENV1–4)	Required	High (real-time thermal cycler, typically laboratory-based)	~3–4 h; longer if transport to centralized lab is required	$$$	High

^*a*^ $, lowest relative cost; $$, intermediate relative cost; $$$, highest relative cost. Relative cost reflects reagents together with equipment/infrastructure requirements; serotype-specific RT-LAMP uses up to four reactions per sample (one per serotype).

The presented dual readout design enables both real-time fluorescence detection and visual endpoint colorimetric detection, rendering the presented assays particularly suitable for use in resource-limited settings. Crucially, both detection modalities rely on indicator dyes incorporated into the reaction mix prior to amplification, allowing results to be read through sealed tubes. This contrasts with LAMP protocols that require post-amplification addition of intercalating dyes, which necessitate opening reaction tubes and pose a significant risk of aerosol-mediated amplicon contamination. The closed-tube format employed here is particularly advantageous for field deployment, where dedicated contamination control infrastructure may be limited or unavailable.

Assay optimization for the portable, battery-operated Axxin T8-ISO isothermal device demonstrates the assays’ suitability for field deployment and robust performance between devices. Interestingly, we could observe a correlation between the fluorescence dye concentration in the reaction and reaction kinetics (data not shown), where reduced fluorescent dye concentration positively affects LAMP reaction speed but can also promote unspecific amplification. This is consistent with reports in the literature and highlights the importance to perform validation and device-specific assay optimization prior to use in a clinical setting (42). As a consequence, the T8-ISO protocol employs a shorter reaction time of 40 min compared to 60 min on laboratory-based real-time fluorimeters, to maintain assay specificity. This run time reduction may affect detection of specimens with low viral loads, particularly for the DENV2 assay where longer amplification times were observed with clinical specimens. However, given that typical acute-phase viremia levels exceed the assay detection thresholds by several orders of magnitude, the impact on clinical detection during the diagnostically relevant febrile window is expected to be limited. Future studies will evaluate the clinical performance of the T8-ISO protocol with clinical specimens to quantify any platform-specific differences in detection rates.

Future developments of DENV RT-LAMP assays should focus on multiplexing capabilities to enable simultaneous detection of all four serotypes in a single reaction, further enhancing the practical utility for surveillance and clinical management. First experiments using a multiplexing approach of the herein designed assays suggest that these assays can be multiplexed to further increase simplicity and reduce costs. Likewise, extended field testing in endemic regions in conjunction with suitable rapid extraction methods will help to ultimately validate performance under real-world conditions.

### Conclusions

Taken together, RT-LAMP assays developed in this study provide a robust, sensitive, and field-deployable solution for DENV detection and serotyping with analytical sensitivities sufficient for detection of acute-phase dengue infections. The combination of broad genetic coverage achieved through strategic introduction of degenerate base positions, dual readout capability in a closed-tube format, clinical validation across multiple serotypes, and successful deployment on a portable isothermal platform distinguishes this work from previous DENV RT-LAMP approaches and represents a practical advancement toward field-deployable dengue serotyping. These assays represent a valuable addition to the diagnostic toolkit for dengue-endemic regions where rapid, accurate, and affordable testing is critically needed and where centralized solutions, like real-time PCR, are not widely available.

## Data Availability

All DENV whole-genome sequences used for primer design were obtained from the NCBI Virus Variation Database (https://www.ncbi.nlm.nih.gov/genomes/VirusVariation/) and are publicly available. RT-LAMP primer sequences are provided in Table 2. Nanopore sequencing reads of LAMP amplicons have been deposited in Zenodo (DOI: 10.5281/zenodo.20933554). Digital PCR quantification data and clinical performance data supporting the findings of this study are provided within the article. Clinical specimen data are not publicly available due to patient privacy and ethical restrictions but are available from the corresponding author upon reasonable request and with approval from the relevant ethics committee.
